# Genome Analyses of the Less Aggressive *Rhizoctonia solani* AG1-IB Isolates 1/2/21 and O8/2 Compared to the Reference AG1-IB Isolate 7/3/14

**DOI:** 10.3390/jof7100832

**Published:** 2021-10-05

**Authors:** Daniel Wibberg, Franziska Genzel, Bart Verwaaijen, Jochen Blom, Oliver Rupp, Alexander Goesmann, Rita Zrenner, Rita Grosch, Alfred Pühler, Andreas Schlüter

**Affiliations:** 1 Genome Research of Industrial Microorganisms, CeBiTec, Bielefeld University, D-33501 Bielefeld, Germany; puehler@cebitec.uni-bielefeld.de (A.P.); aschluet@cebitec.uni-bielefeld.de (A.S.); 2 Institute of Bio- and Geosciences IBG-4, Bioinformatics, Forschungszentrum Jülich GmbH, D-52425 Jülich, Germany; f.genzel@fz-juelich.de; 3 Computational Biology, Faculty of Biology, Bielefeld University, D-33501 Bielefeld, Germany; bverwaai@CeBiTec.Uni-Bielefeld.DE; 4 Bioinformatics and Systems Biology, Gießen University, D-35392 Gießen, Germany; jochen.blom@computational.bio.uni-giessen.de (J.B.); oliver.rupp@computational.bio.uni-giessen.de (O.R.); Alexander.Goesmann@computational.bio.uni-giessen.de (A.G.); 5 Plant-Microbe Systems, Leibniz Institute of Vegetable and Ornamental Crops (IGZ) e.V., D-14979 Großbeeren, Germany; zrenner@igzev.de (R.Z.); grosch@igzev.de (R.G.)

**Keywords:** ultrafast sequencing, AUGUSTUS, GenDBE, EDGAR

## Abstract

*Rhizoctonia solani* AG1-IB of the phylum Basidiomycota is known as phytopathogenic fungus affecting various economically important crops, such as bean, rice, soybean, figs, cabbage and lettuce. The isolates 1/2/21 and O8/2 of the anastomosis group AG1-IB originating from lettuce plants with bottom rot symptoms represent two less aggressive *R. solani* isolates, as confirmed in a pathogenicity test on lettuce. They were deeply sequenced on the Illumina MiSeq system applying the mate-pair and paired-end mode to establish their genome sequences. Assemblies of obtained sequences resulted in 2092 and 1492 scaffolds, respectively, for isolate 1/2/21 and O8/2, amounting to a size of approximately 43 Mb for each isolate. Gene prediction by applying AUGUSTUS (v. 3.2.1.) yielded 12,827 and 12,973 identified genes, respectively. Based on automatic functional annotation, genes potentially encoding cellulases and enzymes involved in secondary metabolite synthesis were identified in the AG1-IB genomes. The annotated genome sequences of the less aggressive AG1-IB isolates were compared with the isolate 7/3/14, which is highly aggressive on lettuce and other vegetable crops such as bean, cabbage and carrot. This analysis revealed the first insights into core genes of AG1-IB isolates and unique determinants of each genome that may explain the different aggressiveness levels of the strains.

## 1. Introduction

*Rhizoctonia solani* Kühn (teleomorph *Thanatephorus cucumeris* [Frank] Donk) belongs to the phylum Basidiomycota and is a soilborne plant-pathogenic fungus that causes diseases in many economically important crops [1]. The anamorph *R. solani* is a species complex including various genetic groups differentiated into anastomosis groups (AGs). Hyphal fusion occurs at high frequency between isolates of the same anastomosis group (AG). Therefore, the ability for hyphal fusion (anastomosis) is used to characterize *R. solani* isolates [2]. To date, based on anastomosis reactions, isolates of the multi-nucleate *R. solani* were classified into 14 AGs, which differ from each other in their behavior [3,4]. Differences include a distinct degree in host specificity of the respective AG, as well as the subdivision of AGs into subgroups. For example, the AG1 comprises several intraspecific subgroups including AG1-IA, AG1-IB, AG1-IC, and AG1-ID, varying in host specificity and pathogenicity [5]. For instance, infection of rice by AG1-IA isolates results in severe sheath blight symptoms, while AG1-IC are non-pathogenic regarding this host plant. Moreover, *R. solani* AG1-IB isolates cause diverse disease symptoms such as sheath blight, leaf blight, foliar blight, root rot, damping-off and bottom rot on a number of agriculturally and horticulturally important crops and plants including corn, sugar beet, rice, common bean, soybean, lettuce, cabbage, figs and hortensia [6]. Previous comparative analyses of available *R. solani* genomes of various pathogenic AGs (AG1-IA, AG1-IB, AG3 and AG8) showed that only 21 to 28% of their genes are shared between all analyzed genomes, thereby representing the first insights into the *R. solani* core genome [7]. Integrated transcriptome analysis between subgroups of AG1, i.e., AG1-IA, AG1-IB and AG1-IC suggested substantial evolutionary distances within AG1, but also common features of isolates belonging to this AG [8]. The importance of the enzymes involved in degrading plant cell walls and expressed during pathogenic interaction with its host plant was shown for AG1-IB [9], as well as for AG3 [10]. Recent studies also reported that phytotoxins such as phenylacetic acid and 3-methylthiopropionic acid are involved in disease symptom development caused by the necrotrophic pathogen *R. solani* [11,12]. Furthermore, it was found that the AG1-IB isolate 7/3/14 encodes much more genes featuring predictable functions in secondary metabolite production, in comparison to the available genomes of the other *R. solani* AGs that were analyzed so far. In particular, RNA-Seq transcriptome data supported previous models of disease progression caused by *R. solani* AGs like bottom rot of lettuce caused by *R. solani* AG1-IB [13], black scurf of potato caused by *R. solani* AG3-PT [10], as well as foliar blight of soybean caused by *R. solani* AG1-IA [14]. Based on the transcriptional responses in these pathosystems, *R. solani* is considered as a necrotrophic plant pathogen. During necrotrophic interaction with host plants, *R. solani* rapidly begins to kill host cells, feeds on these cells and colonizes the surface of the plant. The pathogenic interaction of *R. solani* starts with the detection of the plant, formation of runner hyphea and the formation of infection structures (e.g., infection cushions) to penetrate the plant tissue. It is followed by the secretion of different compounds, including phytotoxins, cell wall degrading enzymes, and other extracellular enzymes affecting host tissue. Genes for products with predicted functions in recognition processes between the fungus and the host plant were identified, as well as genes encoding putative cellulose, pectin and lignin degrading enzymes [10,13]. Furthermore, genes that may play a role in pathogenesis were detected. These most likely specify mitogen-activated protein (MAP) kinase cascades, synthesis of anti-microbial compounds, pyridoxine, unknown effectors, 4-aminobutyric acid (GABA) metabolism, melanin synthesis, plant defence antagonism, phytotoxin, and mycotoxin synthesis [15,16].

The *R. solani* AG1-IB isolate 7/3/14 was isolated from field-grown lettuce plants with bottom rot symptoms. The results of in vivo experiments showed that this isolate caused severe disease symptoms on lettuce, as well as on other vegetable crops, such as bean, cabbage, and carrot [17]. Therefore, this isolate was chosen for the first sequence analysis of an AG1-IB genome and transcriptome [9,13,15,16]. However, in vivo experiments showed that other AG1-IB isolates, obtained from field-grown lettuce plants with bottom rot symptoms, caused only weak symptoms on lettuce compared to the isolate 7/3/14. This observation confirmed that variation in the aggressiveness, the quantitative component of pathogenicity, within a pathogen population exists with the result of differential interactions between the host plant and the pathogen genotype [18]. Evidence for heritability of corresponding features are reported by Pariaud et al. (2009) [19]. 

Therefore, the goal of this study was to establish the first genome sequences of the two less aggressive *R. solani* AG1-IB isolates 1/2/21 and O8/2. In addition, we want to explore intraspecific variation in genome sequences among the three AG1-IB isolates, which differ in levels of aggressiveness. Using the obtained results, we aim to broaden and improve the genomic basis for functional, phylogenetic and comparative analyses focusing on AG1-IB members. In the long-term, the identification of *R. solani* pathogenicity determinants and target-sites for action of biocontrol mechanisms is the focus of our research.

## 2. Materials and Methods

### 2.1. Pathogen Inoculum

The *R. solani* AG1-IB isolates 7/3/14, 1/2/21 and O8/2 were isolated from lettuce plants with bottom rot symptoms and characterized as AG1-IB isolates [17,20]. The isolates were stored on barley kernels at −20 °C. Single barley kernels were placed on potato-dextrose agar (PDA, VWR International GmbH, Germany) and incubated for five days at 20 °C. The inocula for pot experiments were prepared on sterile barley kernels, according to Schneider et al. (1997) [21]. Briefly, sterilized barley kernels were filled in 9 cm Petri dishes and inoculated with mycelial disks (5 mm in diameter in the center) of the respective AG1-IB isolates in the center. Mycelial disks were obtained from an actively growing *R. solani* culture on PDA. The AG1-IB isolates had been grown on the barley kernels for three weeks at 20°C.

### 2.2. Pathogenicity Test

The pathogenicity of the *R. solani* AG1-IB isolates (7/3/14, 1/2/21, O8/2) was tested on lettuce cv. Tizian (Syngenta, Bad Salzuflen, Germany) as described by Chowdhury et al. (2013) [22]. Briefly, the seeds were germinated at 18 °C in a seedling tray, filled with mixture of quartz sand and substrate (Fruhstorfer Einheitserde Typ P, Vechta, Germany) and further cultivated at 20 °C/15 °C 16 h/8 h day/night cycle, 500 µmol m^−2^ s^−1^, 60/80% relative humidity until planting in growth chamber (York, Mannheim, Germany). The seedlings were planted into pots (500 mL), filled with the abovementioned mixture of quartz sand and substrate and cultivated at 22°C/15°C in growth chamber for a further four weeks. One day after planting, each plant was inoculated with two *R. solani*-infested barley kernels in the respective treatments. The infested barley kernels were placed in a distance of 1 cm apart from the lettuce plant. The pots were fertilized weekly (0.2% Wuxal TOP N, Wilhelm Haug GmbH & Co. KG, Düsseldorf, Germany) and lightly watered to maintain the substrate mixture humid. Each treatment included five replicates with six plants per replicate. The impact of the pathogen was determined based on disease incidence (DI, symptoms on stem base), and shoot dry mass (SDM) four weeks after planting because of the previously observed growth inhibition effect caused by *R. solani* AG1-IB on lettuce [22].

The STATISTICA program (StatSoft Inc., Tulsa, OK, USA) was used for data analysis of SDM of lettuce and DI. The SDM and DI data were analyzed using ANOVA with LSD test procedure with *p* ≤ 0.05.

### 2.3. Genome Sequencing and Assembly

Genomic DNA of *R. solani* AG1-IB isolates 1/2/21 and O8/2 was isolated for sequencing on the Illumina MiSeq platform as described previously [15,20,23]. Briefly, to obtain genomic DNA the isolates were cultivated in 60 mL of potato dextrose broth (PDB, SIGMA, Rödermark, Germany) in the dark for five days at 25 °C under low-speed agitation (60 rev min^−1^). The mycelial mats were removed from the broth by filtration and freeze-dried. Genomic DNA was extracted from 50 mg freeze-dried my-celium according to Lee and Taylor (1990) [24] and stored at −20 °C until use. DNA quality and quantity was assessed by gel-electrophoresis, by a fluorescence-based method using the Quant-iT PicoGreen dsDNA kit (Invitrogen) and the Tecan Infinite 200 Microplate Reader (Tecan Deutschland GmbH). In total, 4 μg of purified DNA was used to generate a paired-end library for 1/2/21 and a mate-pair library for O8/2 (Nextera Mate Pair Sample Preparation Kit, Illumina Inc.) for sequencing on the Illumina MiSeq platform. Upon sequencing and processing of the obtained data, the GS De Novo Assembler software (version 2.8) was used for a de novo assembly with heterozygotic mode and default settings. Adapters and low quality reads were removed by an in house software pipeline prior to polishing, as recently described [25].

Benchmarking of assemblies was achieved by using the “3C criterion”—Contiguity, Completeness and Correctness [26]. Contiguity was evaluated by means of QUAST v5.02 [27]. In the next step, completeness was checked by applying BUSCO 3.0.2 [28] with the Basidiomycota single copy gene set 09. All available sequences were mapped to the draft sequences with bowtie2 [29] to check the correctness of the assemblies.

### 2.4. Genome Analysis, Comparative Genomics and Phylogenetics

As described for *R. solani* AG1-IB [15], AG2−2IIIB [25] and AG3-PT [23], a ‘contig-length vs. read-count’ analysis was performed to gain first insights into the genome composition of the less aggressive *R. solani* AG1-IB isolates 1/2/21, and O8/2. The mitochondrial (mt) genomes of the less aggressive *R. solani* AG1-IB isolates were compared to the 7/3/14 mt genome by applying r2cat [30]. In the next step, the eukaryotic gene prediction tool AUGUSTUS (v 3.2.1.) was applied for gene finding [31] by using the established AG1-IB gene model [7]. Predicted genes were annotated within the eukaryotic annotation platform GenDBE [32,33]. Further database comparisons were implemented to improve functional interpretation of the genome sequence, e.g., PhiBase [34], CAZy [35], KAAS [36], and antiSMASH fungal version 6.0 [37]. In the last step, isolates 1/2/21 and O8/2 were compared to the highly aggressive AG1-IB isolate 7/3/14 by means of the comparative genomics tool EDGAR 3.0 [38,39,40]. To calculate the relatedness between the *R. solani* AG1-IB isolates, other *R. solani* strains and the related fungi *Coprinopsis cinerea* okayama 7#130 [41], *Piriformospora indica* DSM 11827 [42], and *Cryptococcus neoformans* var. neoformans JEC21 [43], an average nucleotide identity analysis (ANI), average amino acid identity analysis (AAI), and percentage of conserved proteins (POCP) analysis were performed as described previously [7,23,44,45]. The core genome of all selected fungi was calculated within EDGAR 3.0 and based on all core genes, phylogenetic distances were calculated from multiple sequence alignments. Phylogenetic trees were constructed from concatenated core gene alignments using Fasttree 2.1 [46], as previously outlined in detail [45]. An Euler diagram was created by applying the eulerr R package [47]. Synteny analysis were performed by applying D-GENIES [48]. 

## 3. Results & Discussion

### 3.1. R. solani AG1-IB Isolates 1/2/21 and O8/2 Appeared to Be Significantly Less Aggressive in Pathogenicity Tests in Comparison to AG1-IB 7/3/14

**Figure 1:**
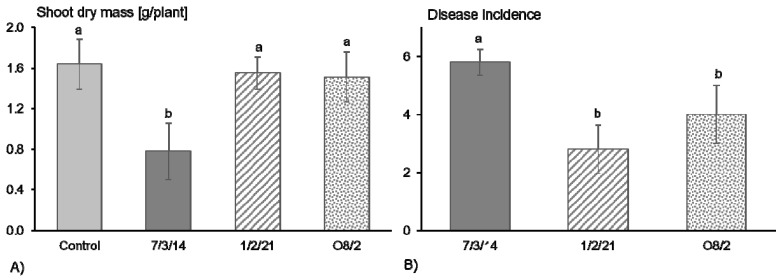
(**A**) Effect of different *Rhizoctonia solani* AG1-IB isolates (7/3/14, 1/2/21, O8/2) on lettuce growth (cv. Tizian) and (**B**) on disease incidence four weeks after pathogen inoculation and cultivation under controlled conditions. Different letters indicate significant differences between treatments according to the least significant difference (LSD) test (*p* ≤ 0.05).

The pathogenicity and in particular aggressiveness of the *R. solani* AG1-IB isolates used in this study were tested based on their impact on lettuce growth. Previous observations demonstrated that infection of lettuce by *R. solani* AG1-IB can result in reduced lettuce biomass [22]. The highest shoot biomass of lettuce was observed in the control treatment (Figure 1A). The isolate 7/3/14 reduced the lettuce growth significantly in contrast to the AG1-IB isolates 1/2/21 and O8/2. On average, around 15% of the lettuce plants shrank after treatment with the isolate 7/3/14. Whereas, no dead plants were observed four weeks after pathogen inoculation by the isolates 1/2/21 and O8/2. Nearly all plants (97%) showed disease symptoms on the stem base in the treatment with the isolate 7/3/14 but only 47% and 67% in the treatments with the isolates 1/2/21 and O8/2, respectively (Figure 1B). These observations confirm that the isolates 1/2/21 and O8/2 are pathogenic but based on the impact on lettuce growth, they appeared to be less aggressive compared to the isolate 7/3/14. Evaluation of the aggressiveness of *R. solani* AG1-IB isolates obtained from the same field highlighted differences between isolates based on results of detached lettuce leaf assays. High aggressiveness was found in around 25% of the tested isolates, whereas the other isolates show weaker aggressiveness [17]. This observation confirms that variation in quantitative pathogenicity within a population exists and results in differences in plant-pathogen interactions. Therefore, differences in the genomes, especially in genes related to pathogenicity, are expected. 

### 3.2. Genome Sequencing of the Less Aggressive R. solani AG1-IB Isolates 1/2/21 and O8/2

The less aggressive AG1-IB isolates 1/2/21 and O8/2 were sequenced in two different sequencing runs on the Illumina MiSeq platform. The sequencing run (2 × 250 bp) of 1/2/21 resulted in 5,264,902 paired end reads yielding approx. 1.3 Gb sequence information representing, on average, a coverage of 30-fold. For O8/2, in total 19,877,036 mate-pair reads were obtained in a second sequencing run resulting in 4.8 Gb of raw data. After processing, about 3.0 Gb sequence information yielded a read coverage of 72-fold. By applying the GS De Novo Assembler software (version 2.8) using the heterozygotic mode, in total 12,854 contigs for 1/2/21 and 19,475 contigs for O8/2 (>500 bp) were generated accounting for a total contig length of 43.8 Mb for 1/2/21 and 49.1 Mb for O8/2 featuring an average GC-content of about 48.30% for both isolates. Based on mate-pair sequence information, assembled contigs of O8/2 were arranged in 1402 scaffolds including 8908 scaffolded contigs. The total assembly size comprising scaffolds larger than 2 kb was calculated to be approx. 41 Mb; the largest scaffold has a size of 2.8 Mb. For 1/2/21, scaffolding was performed based on paired-end sequence information. In total, 2093 scaffolds include 7486 scaffolded contigs with a total length of about 38 Mb. Detailed evaluation of assembly results are shown in Table S1. Obtained values are in the same range as for another completely sequenced AG1-IB isolate, namely 7/3/14 [15,16]. In total, 96.15% (1/2/21) and 93.21% (O8/2) of reads that could be mapped back to the assemblies confirmed the correctness the assembly approaches.

### 3.3. Differentiation of R. solani AG1-IB Isolates 1/2/21 and O8/2 Based on Contig-Length vs. Read-Count Analyses

Based on a ‘contig-length vs. read-count’ analysis, the genomes of *R. solani* AG1-IB isolates 1/2/21 and O8/2 showed a diploid character (Figure S1), which was also the case for the isolate 7/3/14 [15]. The majority of the isolate 1/2/21 and O8/2 contigs (~66%) showed similar coverages that were normalized to 1-fold in this analysis. In contrast to this, approx. 30% of the contigs featured only a 0.5-fold coverage after normalization. The remaining contigs belong to groups with a lower or higher coverage. BUSCO analyses of both strains do not confirm the diploid status, but the completeness of the assembly (BUSCO values for 1/2/21—94.3% - and O8/2—94.6%) indicates that the second line, including chromosomal contigs, harbors only short contigs representing only short fragments of single copy genes. Partial genes on these short fragments were not detected by BUSCO. On the other hand, the single copy genes can be more conserved, and thus found only in the first chromosomal line.

The mitochondrial (mt) genomes of the AG1-IB isolates 1/2/21 and O8/2 were identified based on a comparative approach using the 7/3/14 mt genome as reference. They consist of numerous contigs and show almost no differences compared to the reference. All conserved mt genome regions show a high degree of sequence similarity (> 99%) and comprise all *R. solani* mitochondrial genes. Moreover, the *rrn*-operons of isolates 1/2/21 and O8/2 were also detected in the sequence datasets. Statistically, the *rrn*-operons were present approximately 50 to 60 times in the genomes. This is in the same range as compared to *R. solani* AG2-2IIIB [25,49] and *R. solani* AG3-PT Ben [23]. Whereas, *R. solani* AG1-IB 7/3/14 [15,16] contains around 100 copies of the *rrn*-operon.

### 3.4. Synteny Analysis, Gene Prediction, Annotation and Comparative Genomics Provide First Insights into the R. solani AG1-IB Pan-Genome

To study whether the genetic differences between the different *R. solani* AG1-IB isolates can also be seen in the organization of the chromosomes, a macrosynteny analysis between the three genomes was carried out. In Figure S2, a high genomic synteny can be observed between the three isolates with a few inversions and rearrangements of smaller chromosomal areas. However, due to the amount and size of contigs and scaffolds, larger rearrangements in *R. solani* genomes can be overseen in this analysis.

In the next step, gene prediction for the isolates 1/2/21 and O8/2 was performed. In total, 12.827 (isolate 1/2/21) and 12.973 (isolate O8/2) genes were predicted by means of AUGUSTUS v. 3.2.1. Genes with predicted functions in recognition processes, plant cell wall degradation, and secondary metabolite synthesis were identified in the *R. solani* AG1-IB genome sequences by applying GenDBE (Table 1). The number of predicted genes for the selected categories is in the same range as for the isolate 7/3/14 [15].

**Figure 2:**
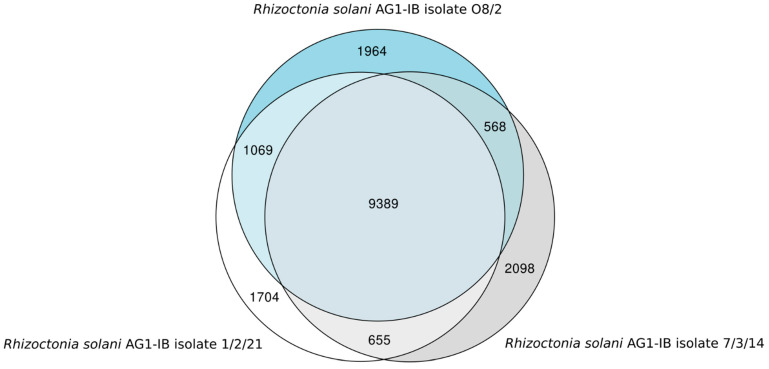
Euler diagram of the gene-based genome comparison for the completely sequenced *R. solani* AG1-IB isolates. The core genome of all isolates consists of 9389 genes. These genes are present in the genomes of all sequenced *R. solani* AG1-IB isolates: O8/2 [EMBL-EBI: CAJZAE010000001-CAJZAE010001402], 1/2/21 [EMBL-EBI: CAJZAD010000001-CAJZAD010002093] and 7/3/14 [EMBL-EBI: CDGK01000001–CDGK01018395 (Contigs); LN679100–LN679996 (Scaffolds)]. For computation of the Venn diagram, default settings of EDGAR 3.0 [34,35,36] were applied.

Based on the comparative genomics tool EDGAR, it appeared that 9389 genes corresponding to approx. 75% of all genes identified in the draft genomes of the two AG1-IB isolates represent the core set of genes shared by all isolates (Figure 2). On the other hand, isolate 1/2/21 possesses 1704 singletons and O8/2 harbors 1964 singletons. Whereas, 2098 singletons were identified for the isolate 7/3/14. Therefore, the *R. solani* AG1-IB pan-genome consists of 17,447 genes.

The singletons of isolates 1/2/21, 7/3/14, and O8/2 were mainly annotated as ‘hypothetical’ genes. However, several singleton genes were assigned to KEGG pathway by applying the KAAS platform. In Table 1, predicted unique gene products of the different *R. solani* AG1-IB isolates are shown, which may play a role in the interaction process with the host plant. Based on this analysis, isolate 7/3/14 appeared to be slightly better equipped regarding interaction with the plant than 1/2/21 or O8/2. It possesses more genes in most of the selected categories than the other isolates. In particular, isolate 7/3/14 encodes considerably more cellulolytic enzymes. However, the differences between the strains are not as pronounced as expected.

In addition to the core genome, the regions involved in secondary metabolite synthesis for the different *R. solani* AG1-IB isolates were analyzed and compared (Table 2). Here, again, the differences between the different strains are not very pronounced. The isolate 1/2/21 has one unique terpene region and O8/2 lacks the siderophore region of 1/2/21 and 7/3/14. All other regions belong to the core genome of the three isolates.

Finally, the isolates 1/2/21 and O8/2 were checked for the most highly expressed and interesting genes recognized in the transcriptome study of Verwaaijen et al., 2017 [13] addressing the analysis of the pathogenic interaction between isolate 7/3/14 and lettuce. Interestingly, all noticeable genes belong to the core genome of the three strains. This demonstrates that the described genes in the mentioned study seem to be essential for the host-pathogen interaction.

In summary, the comparative analyses showed differences between the three isolates. It appeared that isolate 7/3/14 comprises more genes involved in the host-pathogen interaction, which may explain its higher aggressiveness in the pathogenicity tests. However, the differences are not as pronounced as expected.

### 3.5. Phylogenetic Differentiation of R. solani AG1-IB Isolates Based on Shared Core Genes

**Figure 3:**
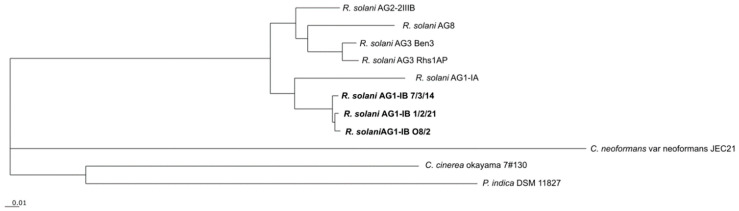
Phylogenetic differentiation of the different *R. solani* AG1-IB isolates. The phylogenetic tree is based on all core genes (473) of the selected strains as determined by means of the comparative genomics tool EDGAR 3.0. The corresponding tree was calculated within EDGAR. It includes *R. solani* AG1-IB (isolates 7/3/14, O8/2, 1/2/21), *R. solani* AG1-IA (isolate B275), *R. solani* AG3 (isolates Rhs1AP, Ben3), *R. solani* AG8 (isolate WAC10335), *R. solani* AG2-2IIIB (isolate BBA 6967), *C. cinerea* okayama 7#130, *P. indica* DSM 11827 and *C. neoformans* var neoformans JEC21. A bootstrap confidence analysis was performed with 1000 replicates to determine the reliability of the tree obtained. All bootstrap values appeared to be 100%.

To deduce the phylogeny of the different *R. solani* AG1-IB isolates in relation to other completely sequenced *R. solani* strains and members of the phylum Basidiomycota, the comparative genomics tool EDGAR was applied. Based on 473 core genes determined for the selected species, a phylogenetic tree was computed (Figure 3).

*R. solani* isolates representing the anastomosis groups AG1-IA, AG1-IB, AG2-2IIIB, AG3 and AG8 cluster together. The *R. solani* AG1-IB isolates build their own cluster in the tree. The other species of the phylum Basidiomycota included in the phylogenetic analysis are only distantly related to *R. solani*. *Coprinopsis cinerea* okayama 7#130 and *Piriformospora indica* DSM 11827 cluster within one group. Whereas, *Cryptococcus neoformans var. neoformans JEC21* is only distantly related to the other fungi. While, *C. cinerea* and *P. indica* belong to the class Agaricomycetes, *C. neoformans* is a member of another class (Tremellomycetes).

To determine the similarities within the *R. solani* AG1-IB species, ANI (Table 3) and AAI values (Table 4) were calculated for these isolates. The obtained results indicate their high sequence similarities. Relatively high ANI values (above 97%) were usually observed for fungal isolates representing the same AG [7], and are considered to indicate species demarcation. Similar results were found for AG3 members [23]. However, the analyzed AG1-IB isolates seem to be more closely related compared to the AG3 isolates.

To better estimate the genomic differences between the selected fungi and to deduce the relative amount of individual genes, a percentage-of-conserved-protein (POCP) analysis was conducted (Table S4) in addition to the calculation of ANI and AAI-values. It should be noted that the results of POCP analyses depend on the reliability of the applied gene prediction models. The more accurate that such models can predict genes and their respective coding sequences, the higher the reliability of POCP results becomes.

To study how POCP results vary across different Basidiomycota classes, *C. cinerea, P. indica* (both Agaricomycetes) and *C. neoformans* (Tremellomycetes) were included in the analyses. The POCP values ranged from 46.1 to 94.7% among the *R. solani* isolates and were in general higher for closely related isolates (according to the results shown in Figure 3). For example, the *R. solani* AG1-IB isolates share 94% of their encoded proteins based on the POCP criteria that are less strict than the EDGAR core genome analysis. Surprisingly, between the selected members of AG1-IB, AG3 and AG2-2IIIB, the POCP is above 80% with one exception (AG2-2IIIB vs. AG1-IB 7/3/14). In comparison, the POCP values between the *R. solani* members of AG1-IA and AG8 range from 46.1 to 66.78%, showing that AG1-IA and AG8 are not that closely related to the other *R. solani* isolates. In this aspect, the POCP analysis underlines the results depicted in the phylogenetic tree (see Figure 3). The POCP values significantly decrease when comparing unrelated species as in the case of *P. indica*, *C. cinerea*, and *C. neoformans*, whereby the values are always below 40%.

The POCP analysis has been proposed as a measure to define genus boundaries in prokaryotes [50]. The authors demonstrated that species of different genera share less than half of their proteins and consequently selected 50% as threshold for genus differentiation. However, as also shown by Wibberg et al. (2020) [45], this value is not applicable for fungal genomes and proves that genes are in general much more conserved in fungi than in prokaryotes. Due to the decrease in POCP values for AG1-IA and AG8, both AGs may represent different genera, while AG1-IB, AG2-2IB and AG3 likely belong to the same genus.

## 4. Conclusions and Outlook

Since the year 2011, the analysis of *R. solani* genomes by high-throughput sequencing technologies and advanced bioinformatics tools for sequence analysis and interpretation has started a new era of studying the evolution, taxonomy, gene content and life style of this form genus. Previously, different *R. solani* genomes were completely sequenced and analyzed, e.g., isolates representing AG1-IA, AG1-IB, AG2-2IIIB, AG3 and AG8.

In this work, additional high quality draft genome sequences of the multinucleate and heterokaryotic fungal species *R. solani* AG1-IB were established that represent less aggressive isolates. *R. solani* AG1-IB isolates 1/2/21 and O8/2 appeared to be significantly less aggressive in pathogenicity tests involving lettuce in comparison to the highly aggressive AG1-IB isolate 7/3/14. Comparative genome analyses revealed first insights into the differences between highly and less aggressive isolates. Unfortunately, it is still not possible to explore the predicted singleton genes of the analyzed isolates by genetic engineering methods since, so far, no robust transformation protocol has been established for *R. solani* AG1-IB.

Based on the core genome analyses from this work in addition to transcriptome sequencing of the isolate 7/3/14, we could show that all significantly higher expressed genes in the different phases during the pathogenic interaction of the fungus and its host plant belong to the core genome of the analyzed AG1-IB members, and thus seem to be essential for their pathogenic interaction.

For future work, the novel long sequence read technologies developed by PacBio and Oxford Nanopore Technologies (ONT) will be used to generate even better fungal genome sequences featuring less contigs and scaffolds. For instance, the ONT technology is relatively inexpensive and especially promising for high GC-content Whole Genome Shotgun sequencing (WGS), given that no PCR amplification is required for the preparation of Nanopore sequencing libraries. In addition, single DNA molecules longer than one megabase can be sequenced using this technology, which will cover longer repeat regions and other regions that cannot be completely resolved by short read methods. Further and improved sequencing of other *R. solani* AG1-IB isolates will also help to determine the pan-genome, the overall degree of genome heterogeneity and to explore rearrangements in this important anastomosis group. Certainly, more fully annotated and interpreted *R. solani* genomic resources are a prerequisite for improvements in disease control strategies.

## Figures and Tables

**Table 1:** Examples of predicted unique gene products for the different *R. solani* AG1-IB isolates.

Selected Groups	1/2/21	O8/2	7/3/14
Celluloytic enzymes ^1^	35	32	46
Tyrosinases ^1^	0	1	3
Drug resistance proteins ^1^	0	0	1
Laccases ^1^	0	0	1
Cytochrome P450 ^1^	7	11	10

^1^ Corresponding genes were listed in Tables S2 and S3.

**Table 2:** Identified secondary metabolite regions for the different *R. solani* AG1-IB isolates.

Secondary Metabolite Regions	1/2/21	O8/2	7/3/14
All	17	15	16
Non-ribosomal peptide synthesis regions	6	6	6
Alkaloid/terpene synthesis regions	10	9	9
Siderophore regions	1	0	1

**Table 3:** Pairwise Average Nucleotide Identity (ANI) analyses for completely sequenced *R. solani* AG1-IB isolates.

ANI	1/2/21	O8/2	7/3/14
1/2/21	100.00	99.12	98.92
O8/2	99.02	100.00	99.02
7/3/14	98.92	99.06	100.00

**Table 4:** Pairwise Average Amino Acid Identity (AAI) analyses for completely sequenced *R. solani* AG1-IB isolates.

AAI	1/2/21	O8/2	7/3/14
1/2/21	100.00	99.46	99.40
O8/2	99.46	100.00	99.46
7/3/14	99.40	99.46	100.00

## Data Availability

The *R. solani* AG1-IB isolates 1/2/21 and O8/2 have been deposited in the DDBJ/EMBL/GenBank database under the Bioproject PRJEB47202 and PRJEB47203 and accession numbers CAJZAD010000001-CAJZAD010002093 and CAJZAE010000001-CAJZAE010001402. The version described in this paper is the first version.

## Supplementary Materials

The following supplementary materials are available online at https://www.mdpi.com/article/10.3390/jof7100832/s1, Figure S1: Contig-length vs. Read-count plots of *R. solani* AG1-IB 1/2/21 and O8/2 contigs. Figure S2: Synteny plots of *R. solani* AG1-IB 1/2/21, O8/2 and 7/3/14. Table S1: Assembly statistcs by means of QUAST, Table S2: Predicted unique CAZyme genes in the *R. solani* AG1-IB genomes, Table S3: Predicted unique genes of *R. solani* AG1-IB genomes, Table S4: Pairwise Percentage of Conserved Proteins (POCP) analysis for *R. solani* strains and selected members of other genera.
