# Supplementary material for: Genome Analyses of the Less Aggressive Rhizoctonia solani AG1-IB Isolates 1/2/21 and O8/2 Compared to the Reference AG1-IB Isolate 7/3/14
Source: J Fungi (Basel). 2021 Oct 5;7(10):832. doi: 10.3390/jof7100832 (PMC8537455; doi:10.3390/jof7100832)
Supplement: Supplementary file 1 [file jof-07-00832-s001.zip › Figure_S2.pdf]

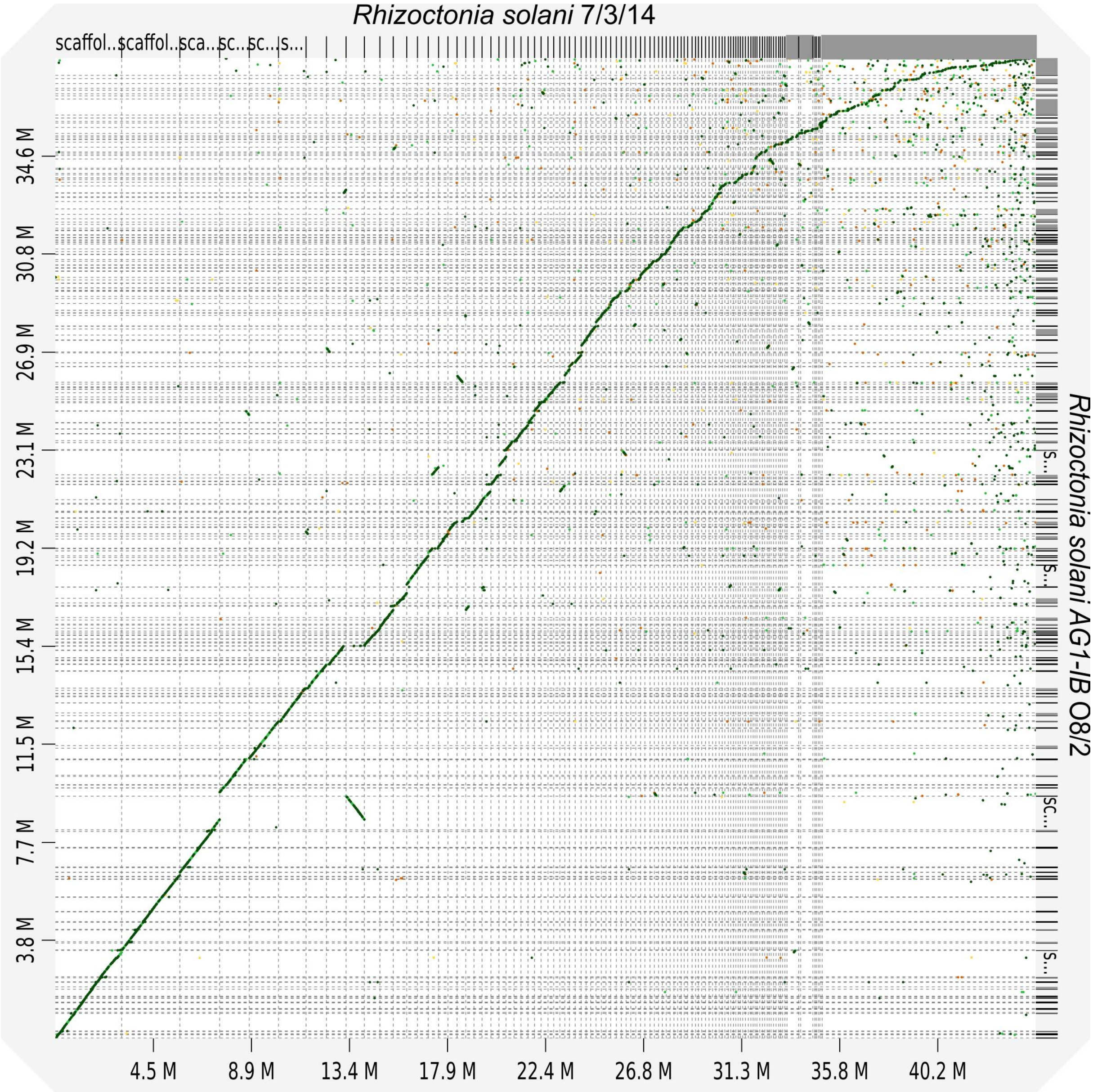

Fig S2a: Comparison of the *R. solani* AG1-IB 7/3/14 genome to the genome of *R. solani* AG1-IB O8/2 by means of D-GENIES.

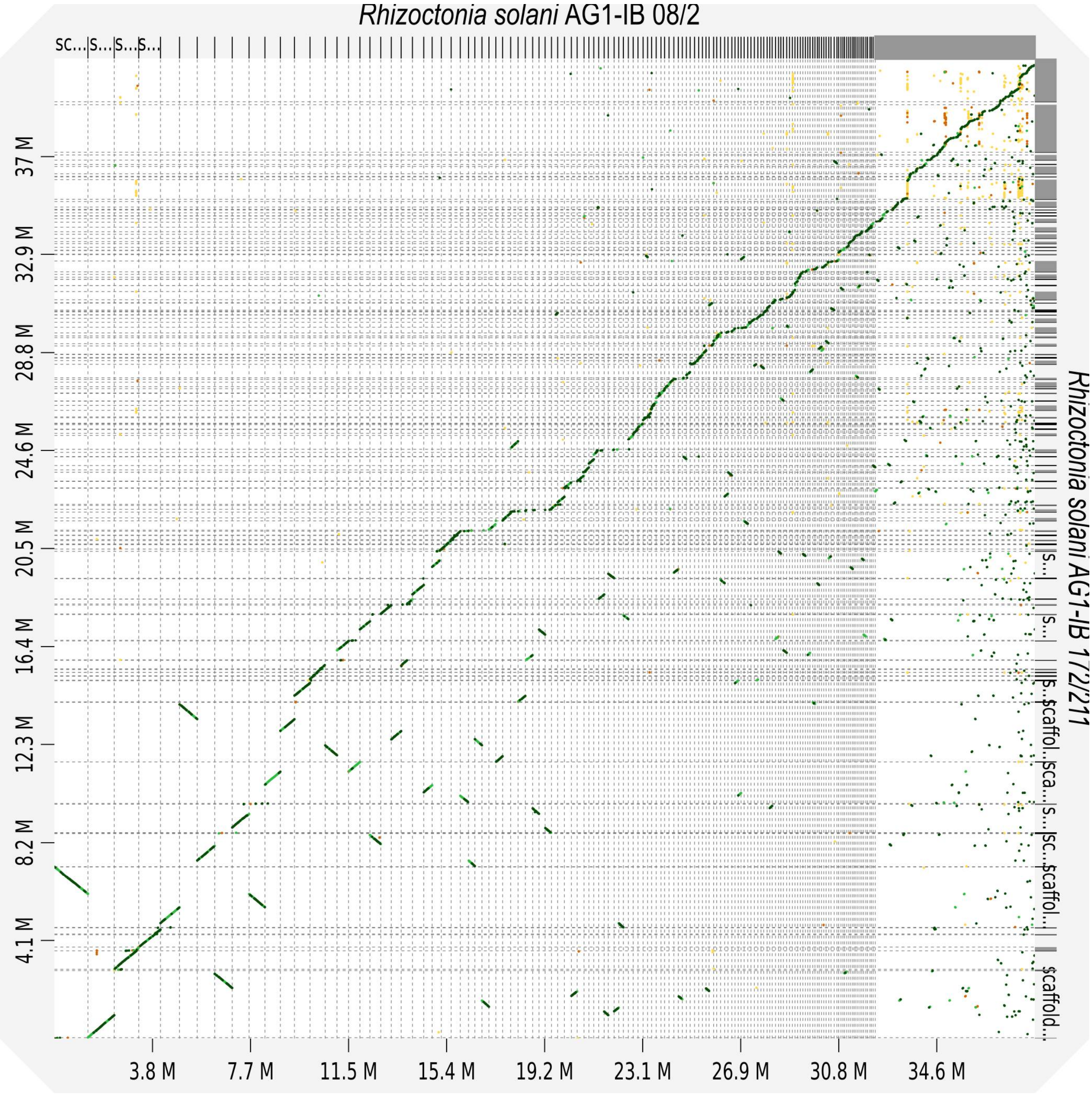

Fig S2b: Comparison of the *R. solani* AG1-IB O8/2 genome to the genome of *R. solani* AG1-IB 1/2/21 by means of D-GENIES.

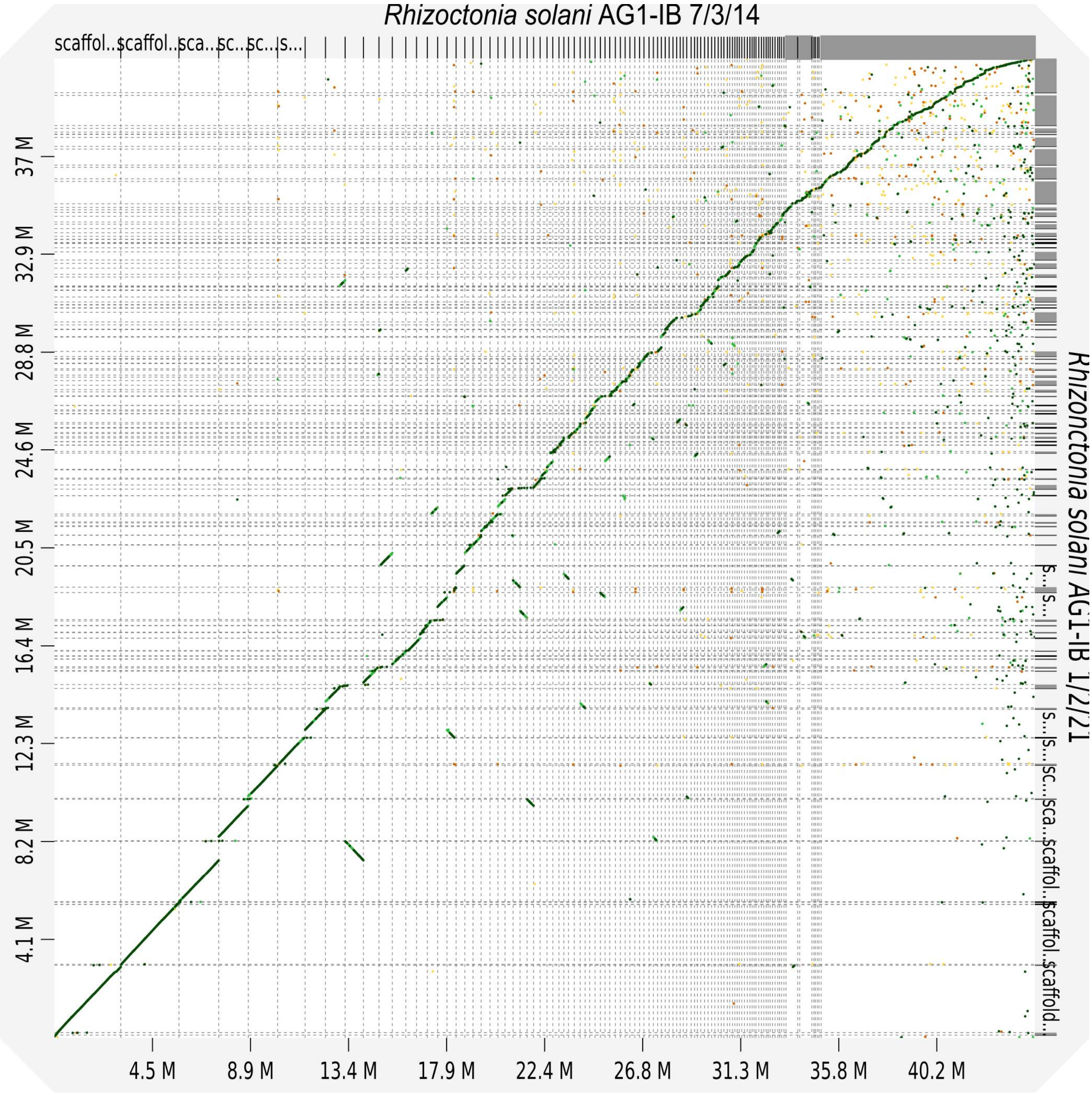

Fig S2c: Comparison of the *R. solani* AG1-IB 7/3/14 genome to the genome of *R. solani* AG1-IB 1/2/21 by means of D-GENIES.
